# Resuscitation training in small-group setting – gender matters

**DOI:** 10.1186/1757-7241-21-30

**Published:** 2013-04-16

**Authors:** Saša Sopka, Henning Biermann, Rolf Rossaint, Steffen Rex, Michael Jäger, Max Skorning, Nicole Heussen, Stefan K Beckers

**Affiliations:** 1 Department of Anaesthesiology, Pauwelsstr. 30, Aachen D–52074, Germany; 2 AIXTRA – Interdisciplinary Centre for Medical Education, Skillslab of the Medical Faculty, Pauwelsstr. 30, Aachen D–52074, Germany; 3 Department of Intensive Care Medicine and Intermediate Care, Pauwelsstr. 30, Aachen D–52074, Germany; 4 Department of Medical Statistics, University Hospital Aachen, RWTH Aachen University, Pauwelsstr. 30, Aachen D–52074, Germany; 5 Section Emergency Medical Care, Department of Anaesthesiology, University Hospital RWTH Aachen University, Pauwelsstr. 30, Aachen D–52074, Germany

**Keywords:** Basic life support (BLS), Cardiopulmonary resuscitation (CPR), External chest compression (ECC), Gender, Training BLS

## Abstract

**Background:**

Within cardiopulmonary resuscitation external chest compressions (ECC) are of outstanding importance. Frequent training in Basic Life Support (BLS) may improve the performance, but the perfect method or environment is still a matter of research. The objective of this study was to evaluate whether practical performance and retention of skills in resuscitation training may be influenced by the gender composition in learning groups.

**Methods:**

Participants were allocated to three groups for standardized BLS-training: Female group (F): only female participants; Male group (M): only male participants; Standard group (S): male and female participants. All groups were trained with the standardized 4-step-approach method. Assessment of participants’ performance was done before training (t1), after one week (t2) and eight months later (t3) on a manikin in the same cardiac arrest single-rescuer-scenario. Participants were 251 Laypersons (mean age 21; SD 4; range 18–42 years; females 63%) without previous medical knowledge. Endpoints: compression rate 90-110/min; mean compression depth 38–51 mm. Standardized questionnaires were used for the evaluation of attitude and learning environment.

**Results:**

After one week group F performed significantly better with respect to the achievement of the correct mean compression depth (F: 63% vs. S: 43%; p = 0.02). Moreover, groups F and S were the only groups which were able to improve their performance concerning the mean compression rate (t1: 35%; t3: 52%; p = 0.04). Female participants felt more comfortable in the female–only environment.

**Conclusions:**

Resuscitation training in gender-segregated groups has an effect on individual performance with superior ECC skills in the female-only learning groups.

Female participants could improve their skills by a more suitable learning environment, while male participants in the standard group felt less distracted by their peers than male participants in the male-only group.

## Background

Well performed external chest compressions (ECC) are of utmost importance for cardiopulmonary resuscitation (CPR) attempts and patient outcome. Adequate external chest compressions (ECC) are necessary during the resuscitation procedure for generating sufficient coronary perfusion pressures in a cardiac arrest situation. If performed as recommended, ECC increase the likelihood of return of spontaneous circulation (ROSC). Thus, ECC are primordial elements of CPR aiming at the promotion of forward blood flow and, therefore, at the maintenance of heart and brain viability. Hemodynamic effects of ECC are dependent on the compression force [1], rate [2] and duration [3]. Inadequate performance of ECC may reduce the success of resuscitation efforts [4,5].

Qualified performance of BLS can only be achieved by sufficient practical training which is mandatory for both laypersons and medical experts. Another important effect is that BLS training increases the willingness to perform BLS in an emergency situation. For example, experience of CPR training is closely associated with willingness to perform bystander CPR [6,7].

Different methods for BLS-training have been established in the past years, while evidence for the best method is rare. Recommendations on the best instructional method are still needed [8,9]. A few models have recently been proposed for training of healthcare professionals [10].

There are clues that composition of learning groups can influence the learning process and outcomes notedly [11]. There is some literature on educational research in gender-divided learner groups in primary and secondary school: Forgasz and Leder have found that girls benefited in sex-single settings with respect to confidence and achievement [12]. In addition, for only boys consisted learner groups was found that co-educational settings (resp. mixed-gender learner groups) seem to be more beneficial for boys [13].

For standard learning situations, the ERC describes the 4-step approach [14,15] as an accepted and feasible compromise. After reviewing the literature we are not aware of recommendations with respect to the composition of adult learning groups as regards the participants’ gender or the learning environment. Retrospectively, our research showed as a side effect that there was a gender influence in training and performing BLS [16].

Therefore, we hypothesized that also in this setting practical performance and retention of skills in learning groups may be influenced by their gender composition.

## Materials and methods

All first-year medical students were enrolled in the present study during their first weeks at the Medical School of RWTH Aachen University, Germany. Before participation, written consent for this investigation was obtained as regards their performance data. The participants were recruited from 251 students who were all laypersons without professional CPR-knowledge, CPR-skills or medical training in this field. Students with any previous medical emergency-training, comparable to emergency medical technician, paramedic, nurse etc., were excluded.

All participants were informed about the study by a standardized hand-out and gave written informed consent. The study was approved by the institutional ethics committee (EK 290/11). Since no potential harm for the participants was expected or even considered possible, the ethics committee asked that informed consent be obtained prior to the study, which was done as described before.

### Study protocol

**Figure 1:**
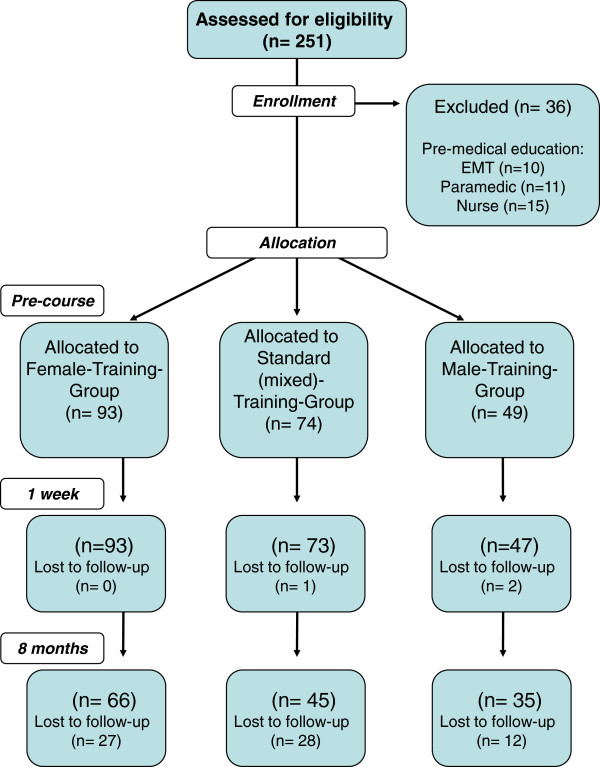
**Study design.** Flow-chart of the study design and distribution of participants according to CONSORT statement.

This three-arm parallel-group trial was conducted at the Department of Anaesthesiology, University Hospital Aachen, Germany in 2008/2009 (Figure 1).

In the first step the allocation of students was conducted by a representative of the Bureau of Student Affairs blinded to the study because of organisational circumstances of the medical school, followed by study group assignment performed by the investigator following the principle of contingency. The allocation procedure resulted in a training group consisting of only female participants (F), a training group consisting of only male participants (M) and a standard training group consisting of male and female participants (S). The baseline testing was provided to ensure that there were no significant differences in the baseline characteristics between the groups.

Prior to the teaching modules, baseline BLS-performance was assessed on a manikin to whom all participants were presented in the same mock scenario in which each individual was asked to resuscitate a person that had just collapsed in front of them. The following standardized text was used:

“Okay, imagine, you are in a public place.

There, this person now collapses without any obvious reason right in front of you.

This manikin represents this person! What are you going to do straight away?

Please take the measures which are necessary in your opinion!”

The performance was followed by no feedback.

Practical test setup was performed with a manikin (Skillreporter Resusci® Anne, Laerdal, Stavanger, Norway) placed in supine position on the floor dressed with a zippered jacket. The procedure was carried out as single-rescuer-CPR (ECC in combination with mouth-to-mouth-ventilation) and terminated at least 180 sec after the first ECC.

After this initial pre-course-assessment, all test persons were taught in the different gender groups and received a standardized BLS-tutorial according to recent resuscitation guidelines [15]. In order to assure consistent instructions for all participants, a standardized and common teaching methodology, the “4-step-approach”, was used [17]. Every participant had the same time to train and the same support during training. Participants were re-tested one week and eight months after the initial BLS-training. Feedback was given only after the third test in order to avoid a bias in performance.

### Measurements, data acquisitions

The manikin was connected to the Laerdal PC Skill Reporting Software (Version 1.3.0, Laerdal, Stavanger, Norway) for data acquisition of ECC. Certified ERC-Advanced Life Support-instructors supervised the performance of each participant and data recording.

Each participant was individually evaluated by a standardized test protocol without being able to recognize other participants’ performance.

### Data analysis

#### Performance data

At the time of performing the study the 2005 guidelines were applied [15], recommending an average compression depth of 38–51 mm and a compression-rate of 90–110 min^-1^. During ECC performance, the compression rate, the numbers of too shallow and too deep compressions were recorded.

### Further data

Besides the practical performance data we recorded demographic data of the participants and the parameter “no delay to start CPR”. Furthermore, we observed the correctness in performance of the algorithm through the parameter “initial assessment”. This parameter was defined as the percentage of participants who performed at least 60% of the BLS-algorithm correctly based on checklist evaluation.

### Questionnaires

Participants completed a standardized pre-course questionnaire before starting the first performance, as well as post-performance questionnaires after the first and the third testing, evaluating subjective learning atmosphere. For this purpose, a 6-point Likert scale was used (from 1 = completely confident to 6 = completely unconfident).

### Statistical analysis

Our primary outcome was the proportion of subjects who achieved targets for compression depth and compression rate. The baseline testing was provided to ensure that there were no significant differences in the baseline characteristics between the groups.

Depending on the scale of the endpoint, two-way repeated measures analysis of variance (repeated measures ANOVA) or two-way repeated logistic regression was carried out to investigate the effect of group (group factor, three levels: F, M, S), time (group factor, 3 levels: baseline, after one week, after 8 months) and the resulting two-factor interaction on group and time. For comparison of effects at a point of time within groups or between time points for one group, suitable contrasts were formulated and tested. Data from the questionnaires was analysed with unpaired t-test to compare differences between groups. Baseline characteristics between groups were inspected by means of unpaired t-test.

Continuous variables were summarized by means and corresponding standard deviations (± SD). Categorical data were presented by frequencies and percentages. All tests were two-sided and assessed at the 5% significance level. Because of the exploratory nature of the parallel study hypotheses no adjustment to the significance level to account for multiple testing was made. The analyses were performed using SAS® statistical software, V 9.2 (SAS Institute, Cary, NC, USA).

## Results

### Study population

From the 251 students starting their medical education, 36 students had to be excluded from the study because of a prior medical education (Figure 1). The groups did not differ with respect to demographic and biometric aspects, about 63% (n = 136) of the tested subjects were female. As demographic data also special variables like age, body height, weighting were recorded (For details refer to Table 1). Further demographic data was comparable between groups: M (n = 49): 21 ± 2 years (range 18–30); F (n = 93): 20 ± 3 years (range 18–40); S (n = 74: 22 ± 4 years (range 18–42).

**Table 1:** Comparison female vs. male group concerning constitution

**Demographics**	Male (M)*(n = 49)	Female (F)*(n = 93)	p-value**
Age	20.8 ± 2.4	20.4 ± 3.1	0.271
Size (cm)	183.1 ± 8.8	170.2 ± 6.5	**0.001**
Body weight (kg)	75.9 ± 12.4	61.4 ± 8.2	**0.001**

*indicated are mean ± standard deviation; **significant differences at a 5%-level are in bold type.

### Observed endpoints

#### Performance data

Prior to the standardized BLS-course (baseline testing), all participants showed poor performance with respect to ECC especially concerning compression rate and compression depth without significant difference between groups (Tables 2 and 3). One week after the standardized BLS-course, all groups improved with respect to an adequate compression depth and compression rate. Regarding the compression depth only the F-group improved significantly (F: 39% to 63%, p = 0.002). The female-only and the standard group improved their compression rate significantly (F: 34% to 51%, p = p = 0.041; S: 25% to 41%, p = 0.0211). Looking at other practical performance parameters one week after training, the female group was significantly better in “compression without faults” than the standard group (Tables 2 and 3), while all groups improved compared to the baseline testing (M: 23(+/−27) to 43(+/−36), p = 0.001; F: 26(+/−30) to 46(+/−33), p = <0.001 S: 20(+/−28) to 34(+/−32), p = 0.003). The male-only group showed significantly more too deep compressions than the other groups, whereas the participants of the standard group performed significantly more frequently too shallow compressions one week after training compared to group F and group M (Tables 2 and 3).

**Table 2:** Overview results performance - baseline and 1 week

	Male (M)*	Female (F)*	Standard (S)*	p-value**
**Baseline (n)**	**46**	**72**	**74**	**M vs. S**
				**F vs. S**
				**M vs. F**
Compression depth 38–51 mm	19 (41%)	28 (39%)	24 (32%)	0.321
				0.449
				0.749
Compression rate 90–110/min	14 (30%)	25 (35%)	19 (25%)	0.575
				0.247
				0.643
No delay to start CPR	23 (50%)	47 (65%)	31 (41%)	0.364
				**0.008**
				0.579
Initial assessment > 60% correct	2 (4%)	4 (5%)	4 (5%)	0.807
				0.976
				0.828
Compression without faults%	23 (±27)	26 (±30)	20 (±28)	0.614
				0.202
				0.543
Compression too shallow%	21 (±32)	34 (±41)	44 (±44)	**0.003**
				0.205
				0.109
Compression too deep%	43 (±41)	27 (±38)	28 (±39)	**0.035**
				0.694
				**0.015**
Compression with incomplete release	8 (±19)	6 (±20)	2 (±8)	**0.039**
				0.131
				0.455
**After 1 week (n)**	**47**	**93**	**73**	
Compression depth 38–51 mm	26 (55%)	59 (63%)*	33 (45%)	0.279
				**0.019**
				0.356
Compression rate 90–110/min.	20 (43%)	48 **(52%)**^**#**^	30 **(41%)**^**$**^	0.899
				0.194
				0.316
No delay to start CPR	38 (80%)	82 (88%)	61 (83%)	0.505
				0.388
				0.246
Initial assessment > 60% correct	36 **(76%)**^**§**^	69 (**74%**)^**§**^	57 **(78%)**^**§**^	0.847
				0.561
				0.759
Compression without faults	43 (±36)	46 (±33)	34 (±32)	0.148
				**0.019**
				0.592
Compression too shallow%	16 (±30)	25 (±33)	46 (±38)	**< .001**
				**< .001**
				0.173
Compression too deep%	33 (±38)	22 (±31)	13 (±28)	**< .001**
				0.076
				**0.036**
Compression with incomplete release	8 (±15)	3 (±12)	2 (±12)	**0.014**
				0.677
				0.027

* indicated are mean ± standard deviation or frequencies with percentages; **significant differences at a 5%-level are in bold type.

Significant differences between groups are indicated **bold**;

Significant differences are concerning time-points:

* p = 0.0016; ^#^ p = 0.0405; ^$^ p = 0.0211; ^§^ p ***<*** 0.0001 (for all groups).

**Table 3:** Overview results performance – 8 months

	Male (M)*	Female (F)*	Standard (S)*	p-value**
**After 8 months (n)**	**35**	**66**	**45**	
Compression depth 38–51 mm	18 (51%)	42 **(64%)**^**@**^	27 **(60%)**^**#**^	0.397
				0.725
				0.216
Compression rate 90–110/min	14 (40%)	34 (52%)^$^	15 (33%)	0.564
				0.064
				0.272
No delay to start CPR	32 (91%)	58 (88%)	40 (89%)	0.663
				0.831
				0.518
Initial assessment > 60% correct	26 **(74%)**^**§**^	43 **(65%)**^**§**^	33 **(73%)**^**§**^	0.920
				0.359
				0.344
Compression without faults	37 (±29)	44 (±35)	41(±34)	0.567
				0.719
				0.341
Compression too shallow	8 (±18)	20 (±30)	20 (±28)	0.109
				0.730
				**0.042**
Compression too deep	43 (±40)	23 (±32)	23 (±34)	**0.005**
				0.858
				**0.005**
Compression with incomplete release	10 (±21)	2 (±11)	2 (±8)	**0.019**
				0.922
				**0.015**

* indicated are mean ± standard deviation or frequencies with percentages; **significant differences at a 5%-level are in bold type.

Significant differences between groups are indicated **bold**;

Significant differences are concerning time-points:

^@^ p=0.0047; ^#^ p=0.0028; ^$^ p=0.0522; ^§^ p***<***0.0001 (for all groups).

As regards retention of skills, group F and the standard group were able to improve their performance regarding compression depth significantly eight months after training (F: 39% to 64%; p = 0. 005; S: 32% to 60%; p = 0.003.. Concerning the compression rate, the group F was the sole group which improved their performance significantly after 8 months (34% to 52%; p = 0.0522). In comparison to the other groups the group F tended to be superior regarding the compression rate (Tables 2 and 3).

### Subgroup analysis

#### Female participants

Comparing the F participants with the female participants from the standard group (SF) reveals a significant difference in performance (F: 63% vs. SF: 43% p = 0.02) one week after training concerning in reaching the correct compression depth. Females of the F-group are still superior in compression depth as well as compression rate compared to those of group S but without statistical significance (Table 4).

**Table 4:** Subgroup-analysis

	**Female only (F)**	**Female standard (S**_**F**_**)**	**Male only (M)**	**Male standard (S**_**M**_**)**	**p-value**
					**F vs. S**_**F**_
					**M vs. S**_**M**_
**Compression depth baseline**	39%	35%	41%	27%	0.664
					0.350
**Compression rate baseline**	34%	27%	30%	23%	0.363
					0.262
**Compression depth after 1 week**	63%	43%	55%	50%	0.02*
					0.811
**Compression rate after 1 week**	52%	43%	43%	36%	0.353
					0.677
**Compression depth after 8 months**	64%	63%	51%	53%	0.973
					0.976
**Compression rate after 8 months**	51%	40%	40%	20%	0.274
					0.858

*significant differences at a 5%-level are in bold type.

### Male participants

Participants from the male-only group compared with male participants of the standard group (SM) at any time point after the training showed a tendency to superior performance in compression depth but at the same time a tendency to be inferior in compression rate. In both cases there was no statistical significance in the difference. Great improvements in compression depth without statistical significance were observed from the male participants in the standard group (for details refer to Table 4).

### Further data

All groups improved significantly in the percentage of “no delay to start CPR” from the pre-course to the one-week-after evaluation. There was no significant difference between the different groups after one week. Eight month after the baseline testing, the female-only group was significantly superior to the standard group in this parameter. In addition, all groups improved their performance in “initial assessment > 60% correct” significantly from the baseline to one week after and to eight months after without any statistical differences between the groups (refer also to Tables 2 and 3).

### Questionnaires

Concerning the question “I felt well with respect to the gender constitution of my training group”, the participants of the male-only groups felt significantly more uncomfortable than the standard group (p = 0.0028). The groups were not different regarding the topic “I would rather train alone”. In fact, the male-only training groups felt significantly more distracted by the group than the other groups (M vs. S p = 0.009; M vs. F p = 0.002) (refer to Table 5).

**Table 5:** Questionnaires concerning learning environment and influence of the group constitution - one week after training

**Questionnaire (one week after training)**	Male (M)*	Female (F)*	Standard (S)*	p-value**
	**(n = 47)**	**(n = 92)**	**(n = 74)**	**M vs. S**
				**F vs. S**
				**M vs. F**
I would rather train alone	5.13 ± 1.08	5.25 ± 0.99	5.11 ± 1.24	0.929
				0.414
				0.533
I felt well with respect of the gender constitution of my group	1.89 ± 0.94	1.61 ± 1.09	1.51 ± 0.71	**0.028**
				0.487
				0.090
I felt distracted by the group	4.91 ± 1.02	5.41 ± 0.84	5.36 ± 0.89	**0.009**
				0.685
				**0.002**

* indicated are mean ± standard deviation; **significant differences at a 5%-level are in bold type.

## Discussion

The presented prospective three-arm trial tested the effects of gender constitution in resuscitation training within a small-group setting for adult laypersons on the individual performance of external chest compression. The main result of this observational study is that one week after the initial resuscitation training, participants of the female-only group showed a significantly better performance with respect to compression depth. Moreover, these groups were able to improve their skills concerning compression rate significantly with training in contrast to the male-only group.

Recent investigations could demonstrate an association between compression depth, the height and weight of children performing BLS [18,19]. However, although our gender-divided groups (F and M) differed significantly with respect to height and weight as expected, there was no significant difference with respect to the proportion of adequately deep compressions. In fact, the male group in our study showed significantly more too deep compressions than the other two groups, a finding observed by other studies as well [20]. Moreover, we couldn’t find evidence that physical properties influence the compression rate performance of adults. Rather, our results indicate that learning success and retention of skills possibly can be attributed to gender-related differences beyond pure physical properties.

Interestingly, participants of the male-only group felt significantly more uncomfortable in their group and more disturbed by the composition of their group than male participants of the “normal” mixed standard group. In contrast, female participants felt more comfortable in single-sex female groups than in mixed groups. These observations correspond to results, which were reported by the analysis of the gender constitution of school classes in which the behaviour of boys was found to frequently alienate girls and to negatively affect girls’ learning success [11]. Several projects of gender- segregated learning groups or environments were initiated due to this observation. They were typically implemented in high-schools or for primary education, but recently also at universities (e.g. the bachelor studies/ Bachelor’s program of informatics and economy at the University of Applied Sciences - Berlin) since the structure of learning groups seems also to influence the learning process [11] of adults. Literature on educational research in gender-divided learner groups in primary and secondary school reports about this interesting fact. Girls benefit from single-sex settings in respect to confidence and achievement as Forgasz and Leder reported before [12] and Colley et al. reports similar findings. Co-educational settings, resp. mixed gender learner groups seem to be more beneficial for male students [13]: Unfortunately no significant literature could be found that describes gender effects in resuscitation training or in real life situations.

One possible explanation why the female-only groups showed a superior performance in compression depth after one week could be the effect of group constitution and learning environment. The post-hoc subgroup analysis showed a significant effect of group affiliation. Superior performance in compression rate seems to be justified by the gender of participants because there is no statistical difference between the performances of the female participants in both groups.

Evidence about the effectiveness of such projects in medical education or acquisition of skills is scarce. Especially there is a lack of literature describing the gender effect in adult learning groups. For resuscitation training, however, we could demonstrate with this study that learning effects of a gender-aligned learning environment for young adults are very interesting. Typically, the retention of skills concerning compression depth after six months or more is on the same level as before training. In our study, female participants from the female-only groups improved their skills after eight months comparing to the baseline. The standard group reached similar performance. Up to now, only few training methods could achieve comparable results [21].

Analysing the male-only group performance, it seems that not only the contentment in this group is less but also their practical performance (e.g. compression rate), at least after one week is not on the same level as in mixed or only female small training groups.

Particularly our results indicate to more suitable learning environments in mixed gender groups. As the participants stated the learning environment felt the best in the female-only group. It seems that the practical performance of females was influenced by this fact. Because it is probably simple and inexpensive to provide gender-related learning environments such an intervention could be a possible method to improve resuscitation skills and knowledge of individual performance through learner groups. On the other hand co-educational settings could be helpful if individuals in male-only learning groups are merely able to reach poor performance in resuscitation skills. From our point the purpose of gender-related training should be only applied to increase the performance of an individual learner through training in a suitable learning environment.

Looking at other skills related to resuscitation efforts, it would be furthermore of interest to analyse if gender-related training environments have any influence on the performance of e.g. airway management or breathing skills in resuscitation settings. Moreover, another interesting issue could be if the performance, knowledge and attitudes of students are influenced by modification of training environments.

Therefore, further studies are warranted that specifically address gender aspects in resuscitation training. For the presented study the 2005 guidelines were applied. The recent 2010 guidelines call for a compression depth of at least 5 cm, which could be associated with different fatigue effects in respect of male or female participants. In future results of this study should be confirmed in a randomized control study with an adequate sample size and adaption of recent guidelines parameters.

### Limitations

Considering possible limitations, we have to admit that entirely our study group represents only a part of the society and a considerable young group of learners. The typical female vs. male ratio in medical schools over Germany is 63% female to 37% male [22]. Thus, in our study we have a representative sample of gender distribution in undergraduate medical schools over Germany. On the other hand this fact causes a smaller number of male participants, which could be considered as limitation as well. Furthermore, the relationship might be different in other countries.

A methodological limitation might be the dropout rate in the eight-month evaluation, for which the main reason was that participants had other appointments (e.g. classes) during the specific evaluation periods. However, the drop-out-rate was comparable in all groups so that a significant bias is not to be expected.

In fact, the applied training situation for gender-related groups is not transferable to real-life emergency situations, because there a specific constitution is not possible. However, this study was not intended to prove this fact but to test the influence of a gender-related training on the performance of an individual learner.

## Conclusions

Our data demonstrate significant effects of gender-segregated training groups on individual practical performance with superior retention of skills after one week in female-only learning groups. Especially the effects on compression rate are interesting at different time points of the study. However, our results possibly indicate that male participants should not be scheduled in male-only settings for resuscitation training in small groups if there are enough female participants to build mixed gender groups. Future training concepts should integrate these aspects adequately.

## Abbreviations

ECC: External chest compressions; BLS: Basic life support; CPR: Cardiopulmonary resuscitation; ERC: European resuscitation council; ROSC: Return of spontaneous circulation.

## Competing interests

All the authors declare that they have no competing interests.

## Authors’ contribution

SSO was responsible for conception and design, acquisition, analysis and interpretation of the data of this study. Finally he was drafting the manuscript. HB was engaged in acquisition of the data and in the critical revision of the manuscript for important intellectual content. RR was involved in the analysis and interpretation of the data and the critical revision of the manuscript for important intellectual content. SR was involved with the analysis and interpretation of the data and a critical revision of the manuscript for important intellectual content. MJ was also responsible for the acquisition and interpretation of the data. MS was involved in the drafting of the manuscript and the critical revision for important intellectual content. NH was responsible for the statistical analysis and the interpretation of the data. Furthermore she was involved in drafting the manuscript. SKB was also responsible for conceptualisation and design of the study concept. He was drafting the manuscript and was involved in the interpretation of the data. Furthermore he revised the manuscript critically and was responsible for the study supervision. All authors read and approved the final manuscript.
